# Generating Large EMF Models Efficiently: A Rule-Based, Configurable Approach

**DOI:** 10.1007/978-3-030-45234-6_11

**Published:** 2020-03-13

**Authors:** Nebras Nassar, Jens Kosiol, Timo Kehrer, Gabriele Taentzer

**Affiliations:** 8 University of Paderborn, Paderborn, Germany; 9 ICREA, Open University of Catalonia, Barcelona, Spain; 10 Philipps-Universität Marburg, Marburg, Germany; 11 Humboldt-Universität zu Berlin, Berlin, Germany

**Keywords:** Model generation, Model transformation, Eclipse Modeling Framework (EMF)

## Abstract

There is a growing need for the automated generation of instance models to evaluate model-driven engineering techniques. Depending on a chosen application scenario, a model generator has to fulfill different requirements: As a modeling language is usually defined by a meta-model, all generated models are expected to *conform to their meta-models*. For performance tests of model-driven engineering techniques, the efficient generation of *large* models should be supported. When generating several models, the resulting set of models should show some *diversity*. *Interactive model generation* may help in producing relevant models. In this paper, we present a rule-based, configurable approach to automate model generation which addresses the stated requirements. Our model generator produces valid instance models of meta-models with multiplicities conforming to the Eclipse Modeling Framework (EMF). An evaluation of the model generator shows that large EMF models (with up to half a million elements) can be produced. Since the model generation is rule-based, it can be configured beforehand or during the generation process to produce sets of models that are diverse to a certain extent.

### 1 Introduction

The need for the automated generation of instance models grows with the steady increase of domains and topics to which model-driven engineering (MDE) is applied. In particular, there is a growing need for large instances of a given meta-model [14,26]. As most of the available MDE tools are based on the Eclipse Modeling Framework (EMF) [34], instances should be conformant to EMF.

Depending on the chosen application scenario, a model generator has to fulfill different requirements: As a modeling language is usually defined by a meta-model, all generated models are expected to *conform to their meta-models*. For

* This work was partially funded by the German Research Foundation (DFG), projects *Generating Development Environments for Modeling Languages (TA294/13-2)* and *Triple Graph Grammars (TGG) 2.0 (TA294/17-1)*.

performance tests of model-driven engineering techniques, the efficient generation of *large* models should be supported. When several models are generated, they should show some diversity. *Interactive model generation* may help in producing relevant models. While there are several tools and approaches to instance model generation in the literature, e.g. [15,16,30,32,36], we are not aware of any tool satisfying all the requirements stated above. Two extreme approaches are the following: The approach in [16] is very fast but does not address any modeling framework and provides very few guarantees concerning the properties of the generated output models. As EMF has developed to the de-facto standard for modeling in MDE, respecting the EMF constraints is crucial to guarantee the usability of the resulting models in practice for processing them by other tools, e.g., for opening them in standard editors. On the contrary, solver-based approaches such as [15,32,36] provide high guarantees by generating instance models that even conform to additional well-formedness constraints (expressed in, e.g., OCL [20]), but they suffer from severe scalability issues.

We suggest finding a good trade-off between having a scalable generation process for models and generating well-formed models. In this paper, we propose a rule-based approach to the generation of models which has the following distinguishing features: (i) To guarantee interchangeability, generated models conform to the standards of EMF. In particular, this means that the containment structure of a generated model forms a tree. (ii) Generated models exhibit a basic consistency in the sense that they conform to the structure and the multiplicities specified by the meta-model. (iii) The generation of models can be configured to obtain models that are diverse to a certain extent. (iv) The implementation is efficient in the sense that instance models with several hundred thousand elements can be generated. (v) The approach is meta-model agnostic and customizable to a given domain-specific modeling language (DSML) in a fully automated way. (vi) It is possible to generate models in a batch mode or interactively to somewhat guide the generation process towards relevant models. User interaction includes the setting of seed models as well as interactively choosing between alternative generation strategies.

Our rule-based approach to model generation consists of two main tasks: (1) The meta-model of a given modeling language is translated into a rule-based model transformation system (MTS) containing rules for model generation. (2) These rules are consecutively applied to generate instance models. This generation process may be further configured by the user. Especially, a potentially inconsistent model may be used as a seed for generating valid models.

Our approach is implemented in two Eclipse plug-ins: A meta-tool, called *Meta2GR*, automatically derives the MTS from a given meta-model. A second plug-in, called *EMF Model Generator*, is automatically configured with the resulting MTS. A modeler uses the configured model generator, which takes additional user specifications and an optional seed EMF model as inputs and generates a valid EMF model. We argue for the soundness of our approach and evaluate its scalability by generating large, valid EMF models (up to half a million elements). Furthermore, we show how to generate a set of models that are diverse to some extent.

### 2 Related Work

In our discussion of related work, we focus on generic approaches and discern between *solver-based*, *tableaux-based* and *rule-based* generic approaches. We omit *language-* and *application-specific approaches* (like, e.g. [7,10]).

#### 2.1 Solver-Based Approaches

Solver-based approaches generate models by (i) translating a meta-model into a logical formula, (ii) using an off-the-shelf solver to find possible solutions, and (iii) translating back the found solutions into instances of the meta-model. In most cases, solver-based approaches are capable of generating models that respect well-formedness constraints such as OCL constraints since these can be translated into the logical formula as well. The approaches presented in [15,32,36] use Alloy [12] for this purpose. Although we do not see any general limitation for them to be applied to arbitrary meta-models, the translations to Alloy presented in [15,36] target dedicated domain-specific languages. The language-independent translation presented by Sen et al. [32] is not fully automated. Performed evaluations show that the scalability of using an off-the-shelf solver is limited to pretty small models.

#### 2.2 A Tableaux-Based Approach

Schneider et al. [27] present an automated approach for the generation of symbolic attributed typed graphs fulfilling a given set of first-order constraints. The approach is based on a tableaux calculus for graph constraints. It produces minimal symbolic models encoding (infinitely) many instances that fulfill the set of constraints. While this is highly desirable to get an overview of possible instance structures, retrieving large graphs from symbolic instances is not directly supported. Moreover, the work does not aim at EMF; it is also not possible to add the EMF constraints as not all of them are first-order. The authors extend their work in [28] to be able to also repair given instances. This model repair can be used to support the generation of instances from a given seed model. The applied repair strategy does not incorporate any deletions of model elements.

#### 2.3 Rule-Based Approaches

Ehrig et al. [9] present an approach for converting type graphs with restricted multiplicity constraints into instance-generating graph grammars. Taentzer generalizes that approach in [37] to arbitrary multiplicity constraints. Both approaches are presented for typed graphs, which means that containment edge types and other EMF constraints are not considered. Moreover, there is no implementation of these approaches.

Radke et al. [24] present a translation of OCL constraints to graph constraints which can be integrated as application conditions into a given set of transformation rules [17]. The resulting rules guarantee validity w.r.t. these constraints but might be rendered inapplicable. The work is motivated by instance generation; however, no dedicated algorithm is presented.

Another grammar-based approach is presented by Mougenot et al. [16]. By reducing models to their containment structure, a tree grammar is derived from that meta-model projection. For a given size (representing the number of nodes), the method is capable of uniformly generating all tree structures of that size. Similarly, the tool *EMF random instantiator* [11] considers containment edges only. While both approaches are highly efficient, reducing models to their containment structure is a severe oversimplification in practice.

The frameworks *RandomEMF* presented by Scheidgen [26] and *EMG* presented by Popoola et al. [23] aid users to manually specify a generator that automatically generates models. These frameworks do not offer any help, however, to ensure that the generated models conform to the meta-model and that the generated models satisfy the required constraints.

The SiDiff model generator (SMG) has been proposed by Pietsch et al. [22]. It takes an existing model as input and manipulates it by applying model editing operations, configured by a stochastic controller. On the meta-level, the SMG was integrated into the approach and tool presented by Kehrer et al. [13,25], which generates a complete set of consistency-preserving edit operations for a given meta-model. It supports meta-models with somewhat restricted multiplicities, however. Generated edit operations can be applied to valid models only. Its stochastic controller has been designed to generate sequences of models that mimic realistic model histories [38]. The generated models are, on purpose, very similar to each other, i.e. they lack diversity.

#### 2.4 A Hybrid Approach

A hybrid approach is implemented within the VIATRA Solver [29,30]: Rules are used to generate an instance model from scratch or a seed model. A solver is used to guarantee validity concerning additional well-formedness constraints. During the generation process, a partial model is extended using rules. This partial model is continuously evaluated w.r.t. the validity of these constraints using a 3-valued logic [31]. By under-approximation, the search space is pruned as soon as the partial model cannot be refined into a valid model. The evaluation of constraints is performed with a specifically developed solver or an off-the-shelf one. All resulting instance models fulfill the additional constraints and conform to EMF. Moreover, the VIATRA Solver has been investigated successfully for generating diverse and realistic models. While experimental results indicate that the approach is 1–2 orders of magnitude better than existing approaches using Alloy, the authors also mention that the scalability of their approach is not yet sufficient [30,29].

**Table 1.** Summary of selected generic approaches to model generation w.r.t. important characteristics we aim at in this paper.

| | | Input | | Output | | Algorithm | | |
|---|---|---|---|---|---|---|---|---|
| Category | Approach | impl. | ex. seed | EMF | wf | config. | interact. | scal. |
| Solver | Sen et al. [32] | + | − | ∘ | + + + | − | − | − |
| Tableaux | Schneider et al. [27,28] | + | ∘ | − | + + + | − | − | ? |
| Rule-based | Taentzer [37] | − | − | − | ++ | ∘ | + | ? |
| | Mougenot et al. [16] | ∘ | − | ∘ | + | ∘ | − | + |
| | Pietsch et al. [22] | + | ∘ | + | + | + | + | ∘ |
| Hybrid | Semeráth et al. [30] | + | ∘ | + | + + + | + | − | ∘ |
| Rule-based | Our approach | + | + | + | ++ | + | + | + |

#### 2.5 Need for Further Research

We summarize the related work through selected approaches from all categories in Table 1 w.r.t. important characteristics. First, we indicate whether the approach is implemented in a tool (column 1). Second, we are interested in manipulating an existing seed model (column 2), e.g., for the sake of generating model evolution scenarios. Here, ∘ indicates that only special kinds of seeds are possible. Third, concerning the consistency level of generated output models, we are interested in the conformance with EMF (column 3) and additional well-formedness constraints, including multiplicities (column 4). Here, + indicates partly and ++ full support of multiplicity constraints, whereas + + + means support of more general well-formedness constraints. Fourth, we are interested in the properties of the generation algorithm itself, which should be configurable (column 5), offer interaction possibilities (column 6), and be scalable (column 7) in order to support the generation of diverse and large instances, respectively.

None of the generic approaches to model generation fully meets all criteria. Given a meta-model with multiplicities as the only well-formedness constraints, we are heading towards a model generator that supports all quality attributes.

### 3 Running Example and Preliminaries

This section presents our running example and preliminaries. After introducing the running example, we recall the Eclipse Modeling Framework (EMF), rule-based model transformation and a rule-based approach to model repair that we utilize for our approach to instance generation.

#### 3.1 Running Example

As running example we use an excerpt of the GraphML meta-model [3] as shown in Fig. 1. GraphML [6] is a file format for different kinds of graphs; it separates

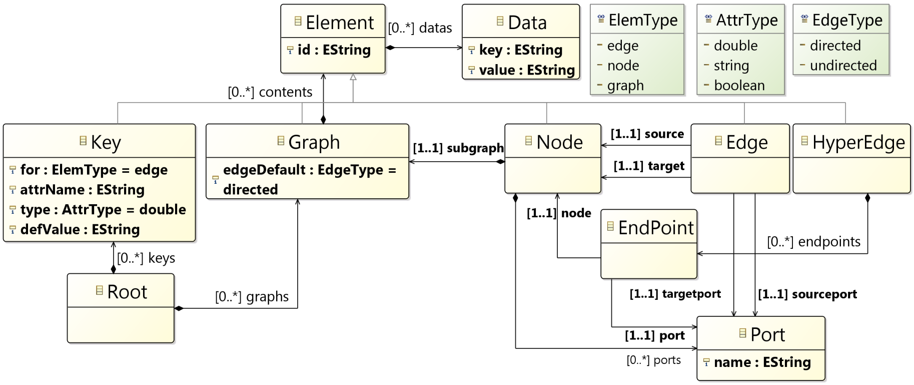

**Fig. 1.** Excerpt of the GraphML meta-model the graph structure from additional data. We use this example to illustrate how our rule-based approach generates instances from a given meta-model.

#### 3.2 The Eclipse Modeling Framework

The Eclipse Modeling Framework (EMF) [34] has evolved into a de-facto standard technology for defining models and modeling languages. In EMF, meta-models are defined using Ecore, an implementation of the OMG's EMOF standard [21]. Meta-models in Ecore prescribe the structures that instance models of the modeled domain should exhibit. Concepts known from UML class diagrams are used, namely the classification of objects and their attributes, references to objects, and constraints on object structures. References may be *opposite* to each other and constrained by *multiplicities*. A specific kind of references are *containments*. The conformance of an instance model to a meta-model can formally be expressed using typed attributed graphs with inheritance [4]. EMF models have to fulfill the following constraints:

– At-most-one-container: Each object must not have more than one container.
– No-containment-cycles: Cycles of containments must not occur.
– No-parallel-edges: There are no two references of the same type from the same source to the same target object.
– All-opposite-edges: If reference types $t1$ and $t2$ are opposite to each other: For each reference of type $t1$, there has to be a reference of type $t2$ linking the same objects in the opposite direction.
– Rootedness (optional): There is an object, called *root object*, that contains all other objects of a model directly or transitively.

In the sequel, we use the terms *EMF model* and *instance model* interchangeably. Each model conforming to its meta-model and fulfilling the EMF constraints listed above is called *EMF model*. If the meta-model's multiplicities are fulfilled in addition, the model is called *valid*. Since we use a graph-based approach to model transformation in the following, objects are often also called *nodes* and object references are called *edges*.

#### 3.3 Transformation Rules and Transformation Units

Our model generation approach is based on the application of *transformation rules* to EMF models as implemented in the Eclipse plug-in Henshin [1,35]. This approach is formally underpinned by typed attributed graph transformation as presented in [4].

A (non-deleting) transformation rule consists of two model patterns, namely a left-hand side $L$ and a right-hand side $R$ where $L$ is a sub-pattern of $R$; we denote such a rule by $L \Rightarrow R$. All elements in $R \setminus L$ shall be created. A rule can be equipped with *negative application conditions* (NACs) [8]. Each NAC $N$ is an additional pattern that includes $L$. All elements in $N \setminus L$ are forbidden to exist. An application of a transformation rule to a model $M$ amounts to finding the pattern $L$ in $M$ and, if such a *match* is found, creating a copy of $R \setminus L$ there. A rule is applicable at a match only if this match cannot be extended to a match for any of the NACs.

In Henshin, rules are specified in an integrated form where elements are annotated and colored according to their roles. While a created element is depicted in green, a forbidden element is shown in blue. Besides, it may be equipped with the name of the NAC it belongs to for distinguishing several NACs. For example, the rule *insert_additionalEdge_targetport* in Fig. 7 matches nodes of types Edge and Port and inserts an edge of type targetport between them but only if such an edge does not already exist and the selected Edge does not already refer to another Port.

To construct more complex transformations in Henshin, rules may be composed in *(transformation) units*. Units may have parameters that can be passed to contained units or rules. A '?' indicates that the parameter may be randomly chosen. We sketch the semantics of those units which we use in the following. Note that each rule is already considered as a unit.

- An independent unit comprises an arbitrary number of sub-units that are checked for applicability in a non-deterministic order. One applicable unit is executed.
- A loop unit comprises one sub-unit and executes it as often as possible.
- A conditional unit comprises either two or three sub-units specifying the if-unit, the then-unit, and optionally, the else-unit. If the if-unit is executed successfully, the then-unit is executed. Otherwise, if defined, the else-unit is executed.
- A sequential unit comprises an arbitrary number of sub-units that are executed in the given order. If a sub-unit is not applicable, it is skipped and the execution continues with the next sub-unit.
- A priority unit comprises an arbitrary number of sub-units that are checked for applicability in the defined order. If a sub-unit is executed successfully, the check and execution of the following sub-units are skipped.

#### 3.4 EMF Repair

Our generation process of instance models uses the repair process for EMF instance models presented in [19]. The basic approach is to derive repair rules from a given meta-model. The derived rules allow to first *trim* the model such that no upper bound is violated any longer. Subsequently, it *completes* the model by adding nodes and edges until no lower bound is violated. The rules are designed such that, during the completion phase, no upper bound violation is introduced and that both phases terminate only if no violation of multiplicities occurs any longer. We formally proved these properties in [18]. While this process does not necessarily terminate, its termination has been proven for instance models of *fully finitely instantiable* meta-models. A meta-model is called *fully finitely instantiable* (f.f.i.) if, for every given finite EMF-model $M$ that instantiates it and respects upper bounds but may violate lower bounds, there exists a finite and valid EMF-model $M'$ such that $M$ is a submodel of $M'$.

### 4 Rule-Based Instance Generation

We start this section with an overview of our approach to the generation of valid EMF models. Thereafter, we present the kinds of generation rules that are derived from a given meta-model, introduce four parametrization strategies for generation processes, and show possibilities of user-interaction. Finally, we discuss the limitations of our generation approach and the formal guarantees that have been shown.

#### 4.1 Overall Approach

Our overall approach to instance generation is depicted in Fig. 2. The fundamental idea behind our approach is to base model generation as far as possible on rule-based model repair using the tool EMF Repair [19]. All rules needed to perform model generation steps are automatically derived from the given meta-model by the meta-tool *Meta2GR*. If a non-empty seed model is given, the model generation process starts with checking it for upper bound violations and potentially trimming it using EMF Repair (*model trimming*). Thereafter, the EMF model is extended with object nodes and references without violating upper bounds using the rules derived by Meta2GR (*model increase*). The resulting model shall meet user specifications w.r.t. its size which will be discussed in more detail in Sect. 4.3 below. In the next step, the EMF model is completed to a valid EMF model, again using EMF Repair (*model completion*). As this repair process adds elements only, the user specifications are still met by the resulting model. Moreover, the result is guaranteed to be a valid EMF model [18]. EMF Repair is also used to set attribute values, either randomly or using user input which is provided in a JSON-file.

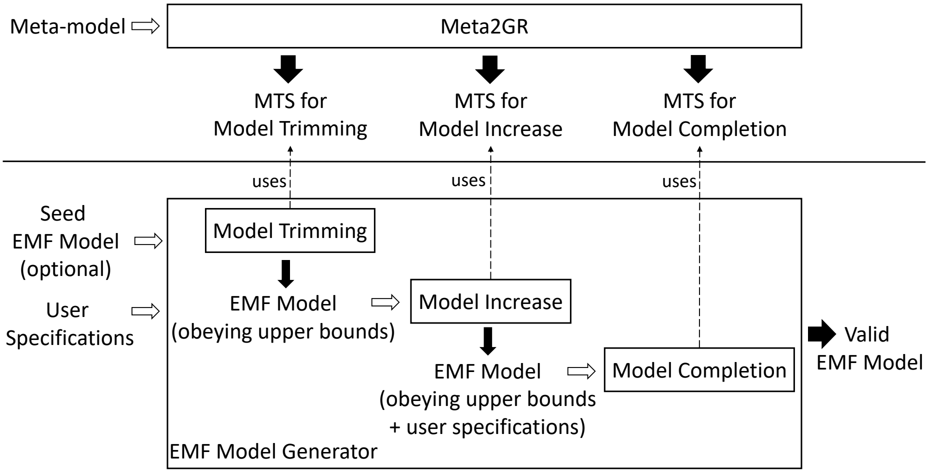

**Fig. 2.** Rule-based EMF Model Generator

#### 4.2 Generated Rules for Model Generation

Given a meta-model, different kinds of rules are derived for generating EMF models. They are listed in Table 2. The derived rules are needed to perform the following tasks: (i) creation of nodes, (ii) insertion of non-containment edges, and (iii) checking for the existence of source or target nodes for an edge of a certain type. All rules that create model elements (i.e., the rules of kinds (i) and (ii)) are generated with NACs to not introduce upper bound violations during generation. Moreover, they all are *consistent transformation rules* in the sense of [4]. This means that they preserve consistency w.r.t. the EMF constraints including rootedness (compare [4, Theorems 1 and 2]). For example, our rules cannot introduce containment cycles or parallel edges by design.

**Table 2.** Overview of rule kinds used for model generation

| Role | Kind | Semantics |
|---|---|---|
| Create node | Additional-node-creation rules | Create a node of a certain type and insert it into one of its direct containers |
| | Transitive-node-creation rules | Create a node of a certain type and insert it into one of its transitive containers |
| Create edge | Additional-edge-creation rules | Create an edge of non-containment type between two nodes |
| Check edge | Additional-edge-checking rules | Check if possible source and target nodes exist for an edge of a certain type |

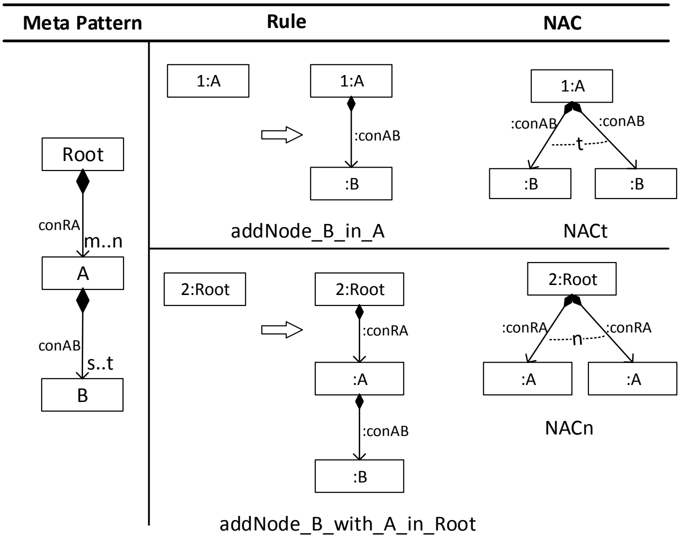

**Fig. 3.** Rule schema for *transitive-node-creation rules* (of length 2)

Node creation (i) is performed by two sets of rules, *additional-node-creation rules* and *transitive-node-creation rules*. The latter ones are described as follows: For every concrete node type in the meta-model, every possible incoming path over containment edges is computed such that each containment type occurs maximally once. For each such path, a rule is derived that matches the node where this path starts and creates the rest of this path. Each rule is equipped with a NAC ensuring that no upper bound violation can be introduced. An example schema of length 2 for this kind of rule is depicted in Fig. 3. The lower part of Fig. 6 depicts all *transitive-node-creation rules* that are derived for the type port. Only one rule is equipped with a NAC as the edge type subgraph is the only one with an upper bound (of 1). In EMF, if a containment edge has an opposite edge, the upper bound of the opposite edge must be 1. If a containment edge is created, the opposite edge is created automatically. Therefore, we do not represent it here. *Additional-node-creation rules* are *transitive-node-creation rules* of length 1. We derive both kinds of rules for different parametrizations of our generation algorithm which are introduced in Sect. 4.3. The rule *add_in_Node_a_Port* in Fig. 6 is an example derived for the containment edge type ports. It does not have a NAC since the upper bound of ports is unlimited.

To create non-containment edges (ii), *additional-edge-creation rules* are generated. The general schema for these kinds of rules is depicted in Fig. 4. For each non-containment edge type, a rule is derived that matches the source and the target nodes suitable to this edge type and creates an edge of the corresponding type. Again, a NAC prevents that an upper bound is violated (NACn). A second NAC prevents that parallel edges are introduced (NACp). If the given edge type has an opposite edge type, the opposite edge is created as well and its upper

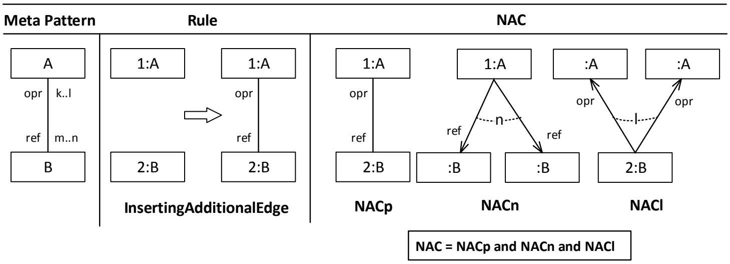

**Fig. 4.** Rule schema for *additional-edge-creation rules*

bound is considered accordingly (NACl). A concrete example for the edge type targetport is the rule *insert_additionalEdge_targetport* as depicted in Fig. 7.

As non-containment edges may be added optionally according to user specifications ( in Sect. 4.3), it is necessary to check if nodes of certain types exist and can serve as source or target nodes of an additional edge without violating the upper bounds of the respective edge type (iii). This check is performed with *additional-edge-checking rules* which are derived for non-containment edge types. The general schema is depicted in Fig. 5. Such a rule is applicable if and only if there exists a source node where the upper bound of the edge type is not yet reached. The same kind of rule is derived for the target node type as well. The rule *check_proper_sourceNode_for_targetport* in Fig. 7 is a concrete example for the edge type targetport.

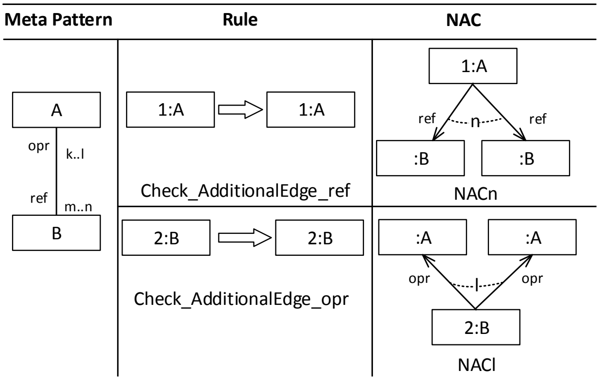

**Fig. 5.** Rule schema for *additional-edge-checking rules*

#### 4.3 Generation Strategies: Parameterization

Since we use a rule-based approach, the model generator can be parameterized w.r.t. a given user specification. In the following, we present four strategies for generating models w.r.t. user specifications; they serve to specify the model increase phase of the generation process. The models resulting from this phase conform to EMF and meet the user specification but may violate lower bounds.

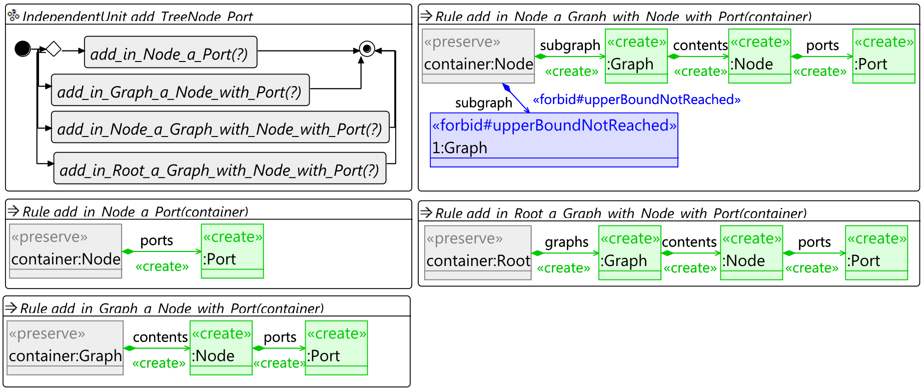

**Fig. 6.** Independent unit for randomly creating a containment tree containing a fixed number of nodes of type Port

They are used as input for the model repair algorithm of EMF repair to obtain a valid EMF model. The user may (1) specify the number of elements that is to be created *minimally*, (2) specify a node type and the number of nodes of this type that is to be created *minimally*, (3) specify an edge type and the number of edges of this type that is to be created *minimally*, or (4) combine the above-mentioned strategies sequentially in arbitrary order. If the user has not specified any model as a seed, the generation is initialized by creating a root node.

**Adding elements of arbitrary types.** In this strategy, the user specifies the minimum of model elements (i.e., nodes and edges) to be created. The idea behind this strategy is to randomly execute a set of rules for adding nodes and edges of arbitrary types without violating the corresponding upper bounds and the EMF constraints. Hence, all rules of kinds *additional-node-creation* and *additional-edge-insertion* are collected into an independent unit which is applied as often as the user specification requires. While the independent unit is implemented in Henshin using a uniform distribution, this strategy may also be performed using other distributions by, e.g., leveraging a stochastic controller [38].

**Adding nodes of a specific type.** In this strategy, the user specifies a node type and the minimum number of nodes of this type that shall be created. This strategy is implemented as an independent unit containing all *transitive-node-creation rules* for the specified node type being applied as often as the user has specified. An example unit for the node type Port is given in Fig. 6.

**Adding edges of a specific type.** In this strategy, the user specifies a (non-containment) edge type and the minimum number of edges that shall be created of this type. This strategy is similar to the previous one, thus its basis is a unit that contains the *additional-edge creation rule* for the specified type. If this rule is not applicable, however, a source or a target node (or both) for an additional edge of that type is missing. The *additional-edge-checking rules* for this edge type are used to detect such situations. Then, corresponding *transitive-node-creation rules* for the type of the missing node are used to create the missing source

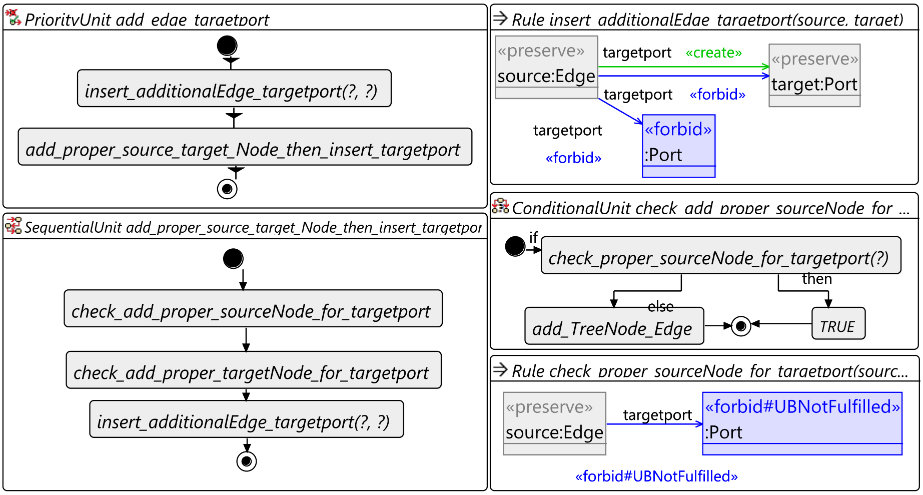

**Fig. 7.** Units for inserting a fixed number of edges of type targetport and/or target node(s). This strategy is implemented as a priority unit where the first contained unit is the *additional-edge-insertion rule*. Its second contained unit is a sequential one with two conditional units checking for missing source or target nodes, respectively, and creating corresponding nodes if needed.

Figure 7 presents a priority unit using this strategy at the example of the targetport-edge. The first level contains the rule *insert_additionalEdge...*. The second level is the sequential unit *add_proper_source_target_Node...*: The conditional unit *check_add_proper_sourceNode...* uses the rule *check_proper_sourceNode...* in the if-statement. The then-statement is set to true whereas the else-statement is configured with a priority unit *add_treeNode_Edge* which adds an Edge-node respecting upper bounds and the EMF constraints. The conditional unit adding a missing target node is defined analogously.

**Sequential combination of strategies.** As our approach allows for an arbitrary seed model as input, the result of applying one strategy can be used as input for applying the second one. This allows for arbitrary sequential combinations of strategies.

### 4.4 User Interaction

Since our approach is rule-based, it is also possible to allow for user interaction. Instead of random rule applications at random matches, the available rules and matches can be presented to the user for selecting at which match a rule has to be applied and how many times. That is promising for generating different tree structures of various weights. While it may not desirable to completely generate large models in such a way, a hybrid strategy can be applied to utilize the selection process, e.g., by employing heuristic data. EMF Repair already supports this kind of user interaction.

#### 4.5 Limitations and Formal Guarantees

*Limitations.* A user may only specify the *minimum* number of desired elements; the specification of a maximum number is not yet supported within our approach. Although the generation process applies the respective rules exactly as often as specified during the model increase phase, some of the rules create more than one element and additional model elements may be created to repair violations of lower bounds during the consecutive model repair. Moreover, we cannot guarantee that the user specification is fully met since necessary rules may not be applicable as often as specified and backtracking is not used. Even if the specification could be met in principle, it may happen that the specific selection, order, and matches of rules do not succeed as they are randomly chosen in the current version of the approach. By counting created elements, it can always be decided whether a user specification has been met, and thus, the user can be informed. In our experiments (in Sect. 6), every generated output meets the selected specifications. Thus, while more research is needed to precisely evaluate the severity of our limitations, the performed experiments are positive evidence that these limitations are rather small even for reasonably complex meta-models.

*Formal guarantees.* In case of termination, our approach guarantees a valid EMF model as output: All generation rules conform to a design that is proven to preserve EMF constraints in [4]. Moreover, applications of these rules cannot introduce violations of upper bounds as they are equipped with corresponding NACs. So each strategy mentioned above is guaranteed to result in an instance model that conforms to EMF and does not violate any upper bounds. Moreover, it is ensured by the finite number of rule calls specified in each strategy that the increase phase terminates. Thus, suitable input for the model completion process of EMF Repair [19] is ensured after finitely many steps. For model completion, termination was proven in the case of f.f.i. meta-models while correctness was proven in all cases in [18]. If the user specification is met after a model has been increased, it is met after model completion as well since no deletion takes place during model completion. Even an increased model that does not meet the user specification is an EMF model and hence a suitable input for EMF Repair. Thus, it can be completed and returned to the user as a valid EMF model. The given user specification, however, is only partly satisfied in this case.

### 5 Tooling

We have developed two Eclipse plug-ins that are available for download.[3] The first plug-in is a meta-tool, called *Meta2GR*. It takes a domain meta-model as input and derives an MTS in Henshin. This is achieved by applying the meta-patterns that are depicted in Figs. 3 to 5 to the given domain meta-model. These meta-patterns are specified as rules typed over the Ecore meta-metamodel. Based on their matches, domain-specific model generation rules of different kinds are

[3] https://github.com/RuleBasedApproach/EMFModelGenerator/wiki created. For a given meta-model, the MTS has to be generated only once. The second Eclipse plug-in, called *EMF Model Generator*, is a modeling tool that uses the derived MTS to generate instance models. Given a user specification and, optionally, one or more seed EMF models, this model generator creates valid EMF models in batch mode or incrementally.

## 6 Evaluation

Next to the formal guarantees which are provided by construction, we empirically evaluate our approach w.r.t. the following research questions:

**RQ 1:** *How fast can instance models of varying sizes be generated?*

**RQ 2:** *Does the use of parametrization help to increase the diversity?*

All experiments were performed on a desktop PC, Intel Core i7, 16 GB RAM, Windows 7 x64 using Eclipse Oxygen. Our Eclipse-based tool was configured to use the default settings, e.g., the heap size was limited to 1 GB. All the evaluation artifacts are available for download.[3]

### 6.1 Scalability Experiments

To answer RQ 1, we conducted two scalability experiments. We used 8 meta-models taken from the literature and projects, namely the Statechart meta-model of Magicdraw [13], Web model [5], Car Rental and Class model [2], Bugzilla, Latex, Warehouse, and GraphML (GML) [3]. The average size of the meta-models is 44 elements (16 nodes, 17 edges, 11 attributes) and the number of multiplicity bounds is 24 on average. The overhead for generating the needed transformation rules and units was, on average, less than 5 seconds, and we will thus focus on the run-time of the model generation in the sequel.

*Experiment 1.* In the first experiment, we randomly generated valid EMF models of varying sizes up to 10 000 elements (counting nodes and edges) for each meta-model using Strategy (1) (in Sect. 4.3). For each size category, we generated 10 valid EMF models and calculated the average run-time. Table 3 presents the results of this experiment. Considering all the meta-models and generated models of varying sizes, our tool always generates a valid EMF model with at least 10 000 elements. Generation times were fastest for the Bugzilla meta-model and slowest for the GraphML one. To assess how robust the times are, we measured the time for generating a seed and for the subsequent repair separately. For each one, we also computed the corrected standard deviation (which is presented for model size 10 000 only). Generating the seed is generally faster than the subsequent repair, except for the StateChart and Warehouse meta-models. If the standard deviation is rather high, this tends to be the case for both, the seed generation and the repair (as for GraphML, Web Model, and Class Model). A closer inspection of the meta-models shows that higher run-times, as well as higher deviations of run-times, are caused by larger meta-model sizes (and hence larger sizes of derived MTSs) and higher numbers of interrelated multiplicity constraints.

**Table 3.** Average run-time (in seconds) for generating valid EMF models of varying sizes for 8 meta-models (MM) using Strategy (1); for size 10 000, run-time is split into the generation of seed and subsequent repair where the corrected standard deviation is added in brackets, respectively.

| MM\Model Size | 1 000 | 3 000 | 5 000 | 8 000 | 10 000 |
|---|---|---|---|---|---|
| Bugzilla | 0.05 | 0.1 | 0.1 | 0.1 | 0.08 (0.006) + 0.04 (0.01) |
| Car Rental | 0.27 | 5 | 17.9 | 72.3 | 65.5 (7.2) + 78.1 (4) |
| Class Model | 0.16 | 1.7 | 9.4 | 61.5 | 13.2 (14.2) + 85 (113.8) |
| CoreWarehouse | 0.81 | 4.5 | 18.9 | 67.9 | 0.4 (0.02) + 131 (10.9) |
| GraphML | 0.4 | 2.6 | 16.7 | 79.2 | 39.3 (56) + 168.1 (119.6) |
| Latex | 1.27 | 1.3 | 1.3 | 1.5 | 0.7 (0.01) + 0.8 (0.03) |
| StateChart | 0.55 | 1.7 | 5.5 | 18.7 | 35.8 (3.9) + 1 (0.3) |
| Web Model | 0.16 | 1.4 | 5.1 | 14.6 | 18.7 (18.8) + 6.2 (2.6) |

**Table 4.** Average run-time and standard deviation (in minutes) for generating valid EMF models of varying huge sizes for the GraphML meta-model using Strategy (3). The standard deviations are presented in brackets.

| Model Size | 200 000 | 300 000 | 400 000 | half a million |
|---|---|---|---|---|
| **Average Time (Min.)** | 6 (1.4) | 11.4 (2.6) | 23.3 (5.7) | 32.5 (6.5) |

*Experiment 2.* The second experiment is dedicated to generating huge models for a complex meta-model which would lead to complex model repair processes. The meta-model GraphML is right for this purpose as its number of lower bounds being non-zero is above the average. Fulfilling these bounds renders model repair into a complex process. We expect the generation of models to become faster when using Strategy (3), i.e., when specifying a minimal number of edge occurrences of a certain type. In this case, nodes are introduced together with incident edges; this generation behavior should reduce the number of repairs needed to take place for fixing lower bound violations. Models of an average size of between 200 000 and 500 000 elements are generated in 6 to 32.5 minutes on average. Each generation process was repeated five times. The standard deviation was between 1.4 to 6.5 minutes, i.e., the run-times for the generation of these huge models are pretty stable. Table 4 presents the experiment results. Moreover, to give an impression of the tool performance for simple meta-models, we applied it to the Bugzilla meta-model. It is considered as simple since it consists of unrestricted containment edges only. The tool needed 1.2 minutes only to generate a valid EMF model with a minimum of 500 000 elements.

#### 6.2 Diversity Experiment

To test if the parametrization of our algorithm has some effect on the diversity of generated models, we conducted the following experiment. We took the GraphML meta-model and chose Strategy (1) to randomly create 10 instance

**Table 5.** Diversity of randomly generated instance models parametrized by node types of the GraphML meta-model (EL = Element, K = Key, etc.; compare Fig. 1)

| | **Str. 1)** | **Str. 2)** | | | | | | | | |
|---|---|---|---|---|---|---|---|---|---|---|
| **Specified Type** | **All** | **EL.** | **K.** | **G.** | **E.** | **H.E.** | **N.** | **P.** | **E.P.** | **D.** |
| **Shannon Index** | 3 | 2.12 | 0.82 | 0.76 | 0.94 | 0.92 | 0.99 | 1.57 | 1.48 | 2.06 |

models containing about 2 000 elements. For each node type as parameter, we created 10 instance models containing about 2 000 elements according to Strategy (2) which specifies that this node type has to occur at least 500 times. For each of the resulting sets of model instances we calculated the Shannon index [33], $\sum_{i=1}^{9} \frac{n_i}{N} \cdot \lg \frac{n_i}{N}$, an established diversity measure. Here, $N$ is the total number of nodes in the given set, $i$ ranges over the 9 non-abstract node types in the GraphML meta-model, and $n_i$ is the number of nodes of that type in the given set. The resulting indices are presented in Table 5. Considering Strategy (1), the types of occurring elements show nearly uniform distribution as the maximal possible Shannon index is $\lg 9 \approx 3.17$. The indices for Strategy (2) show that the distribution of elements significantly differs, depending on the selected node type.

To assess that even the sets with similar Shannon indexes differ from one another, we checked for the types actually occurring in each set and compared them. The results are depicted in Fig. 8. For example, 66 % of the nodes are of type HyperEdge if HyperEdge (H.E.) is chosen as type parameter, and 68 % of the nodes are of type Edge if Edge (E.) is chosen as parameter, even though both sets of models exhibit almost the same Shannon index.

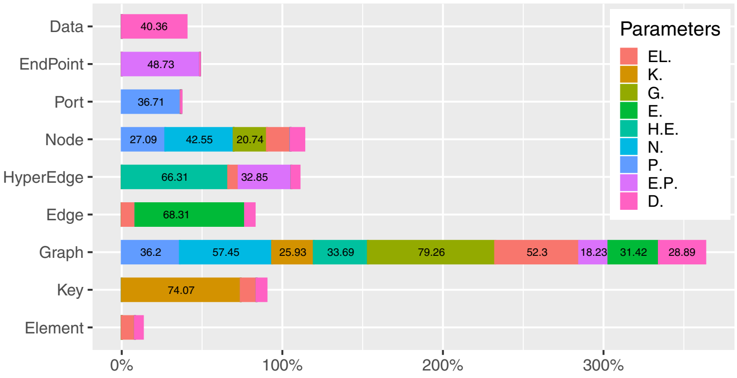

**Fig. 8.** Relative number of occurrences (x-axis) of node types (y-axis) in all the instance models generated using Strategy (2); results obtained for different parameter settings are encoded in colors and each color indicates one instance model. For example, 79.26% nodes of type Graph and 20.74% nodes of type Node are created in an instance model for parameter Graph (G.).

To answer RQ 2, choosing different node types as parameter leads to significantly different distributions of the node types of occurring elements. Hence, Strategy (2) can be used to introduce a certain diversity.

#### 6.3 Threats to Validity

In our evaluation, we selected 8 meta-models. Evaluation results might differ when choosing others. We are confident, however, that our results are representative as we selected meta-models from diverse backgrounds, with reasonable sizes, and with varying numbers and forms of multiplicities. The used metric to measure diversity completely abstracts from details of the underlying graph structures of generated instance models. On the one hand, abstracting from such details typically underrates diversity rather than overrating it. On the other hand, we have to acknowledge that the form of diversity we show in our experiments is limited to the distribution of types.

### 7 Conclusion and Future Work

We developed a rule-based approach for generating valid models w.r.t. arbitrary multiplicities and EMF constraints. Since we use a rule-based approach, our generator is configurable to support user specifications and to allow user interaction. Several parameterization strategies are presented to generate different sets of valid EMF models. Two Eclipse plug-ins have been developed: *Meta2GR* automatically translates the meta-model of a given DSML to an MTS and the *EMF Model Generator* uses the derived MTS to generate valid EMF models. We evaluated the scalability of our approach by generating large instances of several meta-models of different domains and showed that models with 10 000 elements can be generated in about a minute on average. Furthermore, our tool can generate valid EMF models of 500 000 elements in less than 2 minutes for a meta-model with largely unrelated multiplicity constraints and in about 30 minutes for a meta-model with closely interrelated ones. Moreover, we showed that a certain form of diversity between the generated models can be achieved by configuration. As future work, we intend to support meta-models with OCL constraints, at least partly: Integrating the constraints as application conditions into rules [17,24] is a promising basis to extend our approach in this direction. Besides, we want to support further configuration facilities which allow us to generate realistic models by leveraging a stochastic controller [38].

## References

[1] Arendt, T., Biermann, E., Jurack, S., Krause, C., Taentzer, G.: Henshin: Advanced Concepts and Tools for In-Place EMF Model Transformations. In: Proc. MODELS. pp. 121–135. Springer (2010)

[2] Arendt, T., Taentzer, G.: A tool environment for quality assurance based on the eclipse modeling framework. Automated Software Engineering **20**(2), 141–184 (2013)

[3] Atlantic Zoo. http://web.imt-atlantique.fr/x-info/atlanmod/index.php?title=Zoos (2019)

[4] Biermann, E., Ermel, C., Taentzer, G.: Formal Foundation of Consistent EMF Model Transformations by Algebraic Graph Transformation. SoSyM **11**(2), 227–250 (2012)

[5] Brambilla, M., Cabot, J., Wimmer, M.: Model-Driven Software Engineering in Practice. Morgan & Claypool Publishers (2012)

[6] Brandes, U., Eiglsperger, M., Herman, I., Himsolt, M., Marshall, M.S.: GraphML Progress Report: Structural Layer Proposal. In: Graph Drawing. pp. 501–512. Springer (2002)

[7] Brottier, E., Fleurey, F., Steel, J., Baudry, B., Le Traon, Y.: Metamodel-based test generation for model transformations: an algorithm and a tool. In: Symp. on Software Reliability Engineering. pp. 85–94 (2006)

[8] Ehrig, H., Ehrig, K., Prange, U., Taentzer, G.: Fundamentals of Algebraic Graph Transformation. Springer (2006)

[9] Ehrig, K., Küster, J.M., Taentzer, G.: Generating instance models from meta models. SoSyM **8**(4), 479–500 (2009)

[10] Fleurey, F., Steel, J., Baudry, B.: Validation in model-driven engineering: testing model transformations. In: Proc. Intl. Workshop on Model, Design and Validation. pp. 29–40. IEEE (2004)

[11] Gómez, A., AtlanMod Team: EMF random instantiator (2015), https://github.com/atlanmod/mondo-atlzoo-benchmark/tree/master/fr.inria.atlanmod.instantiator, (visited on 2020-02-18)

[12] Jackson, D.: Alloy: A lightweight object modelling notation. ACM Trans. Softw. Eng. Methodol. **11**(2), 256–290 (2002)

[13] Kehrer, T., Taentzer, G., Rindt, M., Kelter, U.: Automatically Deriving the Specification of Model Editing Operations from Meta-Models. In: Proc. ICMT. pp. 173–188 (2016)

[14] Kolovos, D.S., Rose, L.M., Matragkas, N., Paige, R.F., Guerra, E., Cuadrado, J.S., De Lara, J., Ráth, I., Varró, D., Tisi, M., et al.: A research roadmap towards achieving scalability in model driven engineering. In: Workshop on Scalability in Model Driven Engineering. ACM (2013)

[15] McGill, M.J., Stirewalt, R.K., Dillon, L.K.: Automated test input generation for software that consumes ORM models. In: OTM Confederated Intl. Conferences. pp. 704–713. Springer (2009)

[16] Mougenot, A., Darrasse, A., Blanc, X., Soria, M.: Uniform random generation of huge metamodel instances. In: European Conf. on Model Driven Architecture-Foundations and Applications. pp. 130–145. Springer (2009)

[17] Nassar, N., Kosiol, J., Arendt, T., Taentzer, G.: OCL2AC. Automatic Translation of OCL Constraints to Graph Constraints and Application Conditions for Transformation Rules. In: Proc. ICGT 2018. pp. 171–177. Springer (2018)

[18] Nassar, N., Kosiol, J., Radke, H.: Rule-based Repair of EMF Models: Formalization and Correctness Proof. In: Electronic Pre-Proc. Intl. Workshop on Graph Computation Models (2017)

[19] Nassar, N., Radke, H., Arendt, T.: Rule-based repair of EMF models: An automated interactive approach. In: Proc. ICMT. pp. 171–181 (2017)

[20] OMG: Object Constraint Language. (2014), http://www.omg.org/spec/OCL/

[21] OMG: OMG Meta Object Facility (MOF). Version 2.5.1 (11 2016), http://www.omg.org/spec/MOF/

[22] Pietsch, Pit and Yazdi, Hamed Shariat and Kelter, Udo: Generating realistic test models for model processing tools. In: Proc. ASE. pp. 620–623. IEEE CS (2011)

[23] Popoola, S., Kolovos, D.S., Rodriguez, H.H.: EMG: A domain-specific transformation language for synthetic model generation. In: Proc. ICMT. vol. 9765, pp. 36–51. Springer (2016)

[24] Radke, H., Arendt, T., Becker, J.S., Habel, A., Taentzer, G.: Translating Essential OCL Invariants to Nested Graph Constraints for Generating Instances of Meta-models. Science of Computer Programming **152**, 38–62 (2018)

[25] Rindt, M., Kehrer, T., Kelter, U.: Automatic generation of consistency-preserving edit operations for mde tools. Demos @ MoDELS **14** (2014)

[26] Scheidgen, M.: Generation of large random models for benchmarking. In: BigMDE@ STAF. pp. 1–10 (2015)

[27] Schneider, S., Lambers, L., Orejas, F.: Automated reasoning for attributed graph properties. Intl. Journal on Software Tools for Technology Transfer **20**(6), 705–737 (2018)

[28] Schneider, S., Lambers, L., Orejas, F.: A logic-based incremental approach to graph repair. In: Fundamental Approaches to Software Engineering. pp. 151–167. Springer (2019)

[29] Semeráth, O., Babikian, A.A., Pilarski, S., Varró, D.: Viatra solver: a framework for the automated generation of consistent domain-specific models. In: Proc. ICSE. pp. 43–46. IEEE/ACM (2019)

[30] Semeráth, O., Nagy, A.S., Varró, D.: A Graph Solver for the Automated Generation of Consistent Domain-specific Models. In: Proc. ICSE. pp. 969–980. ACM (2018)

[31] Semeráth, O., Varró, D.: Graph constraint evaluation over partial models by constraint rewriting. In: Proc. ICMT. pp. 138–154 (2017)

[32] Sen, S., Baudry, B., Mottu, J.M.: Automatic model generation strategies for model transformation testing. In: Proc. ICMT. pp. 148–164 (2009)

[33] Shannon, C.E.: A Mathematical Theory of Communication. SIGMOBILE Mob. Comput. Commun. Rev. **5**(1), 3–55 (2001), reprint

[34] Steinberg, D., Budinsky, F., Paternostro, M., Merks, E.: EMF: Eclipse Modeling Framework. Addison Wesley, Upper Saddle River, NJ, 2 edn. (2008)

[35] Strüber, D., Born, K., Gill, K.D., Groner, R., Kehrer, T., Ohrndorf, M., Tichy, M.: Henshin: A Usability-Focused Framework for EMF Model Transformation Development. In: Proc. ICGT. pp. 196–208 (2017)

[36] Svendsen, A., Haugen, Ø., Møller-Pedersen, B.: Synthesizing software models: generating train station models automatically. In: Intl. SDL Forum. pp. 38–53. Springer (2011)

[37] Taentzer, G.: Instance generation from type graphs with arbitrary multiplicities. ECEASST **47** (2012)

[38] Yazdi, H.S., Angelis, L., Kehrer, T., Kelter, U.: A framework for capturing, statistically modeling and analyzing the evolution of software models. Journal of Systems and Software **118**, 176–207 (2016)

